# Root hair-specific transcriptome reveals response to low phosphorus in *Cicer arietinum*

**DOI:** 10.3389/fpls.2022.983969

**Published:** 2022-10-04

**Authors:** Pawandeep Singh Kohli, Lekha T. Pazhamala, Balaji Mani, Jitendra Kumar Thakur, Jitender Giri

**Affiliations:** ^1^National Institute of Plant Genome Research (NIPGR), New Delhi, India; ^2^International Center of Genetic Engineering and Biotechnology, New Delhi, India

**Keywords:** root hair, phosphate, gene expression, chickpea, legumes, RNA-seq

## Abstract

Root hairs (RH) are a single-cell extension of root epidermal cells. In low phosphorus (LP) availability, RH length and density increase thus expanding the total root surface area for phosphate (Pi) acquisition. However, details on genes involved in RH development and response to LP are missing in an agronomically important leguminous crop, chickpea. To elucidate this response in chickpea, we performed tissue-specific RNA-sequencing and analyzed the transcriptome modulation for RH and root without RH (Root-RH) under LP. Root hair initiation and cellular differentiation genes like RSL TFs and ROPGEFs are upregulated in Root-RH, explaining denser, and ectopic RH in LP. In RH, genes involved in tip growth processes and phytohormonal biosynthesis like cell wall synthesis and loosening (cellulose synthase A catalytic subunit, *CaEXPA2*, *CaGRP2*, and *CaXTH2*), cytoskeleton/vesicle transport, and ethylene biosynthesis are upregulated. Besides RH development, genes involved in LP responses like lipid and/or pectin P remobilization and acid phosphatases are induced in these tissues summarizing a complete molecular response to LP. Further, RH displayed preferential enrichment of processes involved in symbiotic interactions, which provide an additional benefit during LP. In conclusion, RH shows a multi-faceted response that starts with molecular changes for epidermal cell differentiation and RH initiation in Root-RH and later induction of tip growth and various LP responses in elongated RH.

## Introduction

Low soil phosphorus (P) conditions pose a significant hurdle for optimum plant growth and crop yield. The problem is further compounded by the high rate of P fixation in the soil in the form of insoluble complexes. Therefore, to acquire water-soluble inorganic phosphate (Pi) from the soil, various morphological changes occur in the root architecture of plants growing in low P soils (Péret et al., 2011). The most widespread changes observed in root architecture are longer and denser laterals and root hairs (RH), which facilitate a larger surface area for P acquisition (Bates and Lynch, 1996; Keyes et al., 2013; Heppell et al., 2015). Both RH and laterals contribute to soil exploration and P acquisition; however, being a single-cell extension of the epidermal cell, RH put lower carbon cost, not laying energy penalty on the plant compared to laterals (Lynch and Ho, 2005; Gonzalez et al., 2021; Marin et al., 2021). Further, RH contributes to nearly half of the total P acquisition and could indirectly affect P availability through their diverse emerging roles in the rhizosphere, like plant-microbe interaction and modulating soil structure (Keyes et al., 2013; Kohli et al., 2021). Thus, RH traits could be promising targets for maintaining P acquisition and yield stability in low P soil fields.

The molecular details of RH’s developmental response to low P (LP) are very well understood in the model plant, Arabidopsis. Local sensing of LP in the soil leads to auxin accumulation in lateral root cap cells and xylem (Bhosale et al., 2018; Wendrich et al., 2020). The auxin concentration in vascular tissue and epidermal files is maintained through transporters like AUX1 and local auxin biosynthesis, facilitating the LP response (Bhosale et al., 2018). Accumulated auxin in the xylem induces cytokinin biosynthesis through TMO5/LHW transcription factor (TF) complex. Here, cytokinin acts as a mobile signal leading to increased RH density (Wendrich et al., 2020). LP conditions activate both auxin and ethylene signaling, which induce RH developmental-related genes, especially the TF RHD Six-Like 4 (RSL4; Datta et al., 2015; Feng et al., 2017; Bhosale et al., 2018). RSL4 is directly involved in inducing RH cell developmental genes like cell-wall organization and synthesis, cellular membrane development, cytoskeleton, vesicular transport, and reactive-oxygen species regulation leading to induction in tip growth (Vijayakumar et al., 2016).

Apart from Arabidopsis, in model crop species like rice, maize, and soybean, similar genes and processes are known to be involved in RH cell development. Characterization of *osaux1* mutant in rice reveals a similar role of AUX1 in LP response as in Arabidopsis (Giri et al., 2018). Also, various RH development mutants are known and have been characterized in maize and soybean (Jung and Schnable, 1994; Klamer et al., 2019; Yang et al., 2019). Other than mutant characterization, tissue-specific transcriptomic profiling revealed essential RH development genes and probable functions of RH. In maize and rice, RH-specific transcript profiling identified various genes exclusive and preferential to RH and has potential roles in RH cell development (Hey et al., 2017; Moon et al., 2018). Further, RH-specific transcriptome under external stimuli like Nod factors, symbionts, and cadmium toxicity depict involvement of RH in rhizobia infection, symbiosis, and mineral toxicity (Libault et al., 2010; Damiani et al., 2016; Cao et al., 2019). RH response to LP is well characterized by single-cell RNA-seq in Arabidopsis (Wendrich et al., 2020). These tissue-specific transcriptomes for RH are limited to a few model crop species, and profiling of RH-specific and preferential genes in various other crops like chickpea is still lacking. Therefore, in the present study, we performed RH-specific transcriptome in an economically important legume chickpea under normal phosphorus (NP) and LP conditions to identify genes and responses preferential to RH and involved in RH development and various other processes.

Chickpea is one of the most nutritious pulses and a vital source of protein for South Asia. In India, chickpea holds a share of 46% of total pulse production (Anon, 2018). However, a considerable area for chickpea production lies under poorly fertilized and nutrient-poor lands with limited P availability, posing a major constraint for achieving optimum yield (Srinivasarao et al., 2006). Therefore, it is crucial to develop chickpea varieties with better P acquisition and soil exploration capacity. For this, it is critical to understand and characterize the response of rooting structures like laterals and RH during LP in chickpea. With this objective, we have identified genes differentially expressed in LP in RH and Root without RH (Root-RH) under LP. The transcriptome landscape revealed critical processes and regulations enriched during LP in specific tissues. We have also identified specific and preferential genes expressed in RH of chickpea, revealing enriched processes preferentially occurring in RH.

## Materials and methods

### Plant material and growth conditions

A chickpea (*Cicer arietinum*) cultivar, “ICC4958,” was used to study the transcripts for the early and local response to phosphate (Pi) deficiency in RH and Root-RH. Two sets of surface-sterilized seeds were germinated on Hoagland media with phosphate sufficient (NP—Normal Phosphorus; 252.1 μM NaH_2_PO_4_.2H_2_O) and phosphate deficient (LP—Low Phosphorus; 0 μM NaH_2_PO_4_.2H_2_O) conditions, respectively, as described in Kohli et al. (2020). The roots of 12-day-old seedlings were flash-frozen in liquid nitrogen to harvest the RH and Root-RH samples, as described in Li et al. (2016). Two biological replicates, each containing 120 seedlings, were used to collect RH by scrapping the roots (Supplementary Figures 1A,C). After scrapping, the root component was used for the Root-RH sample (Supplementary Figure 1B).

### Phenotyping for root hair and root traits

Twelve days old chickpea seedlings were phenotyped for RH and root traits. Root traits like primary root length, lateral root number, and density were measured using a measuring scale. For RH traits, roots were imaged using a stereo zoom microscope (Leica S9i, Germany), and images were processed using ImageJ software to measure RH length and distance from tip to first visible RH. Average root diameter was measured using ImageJ software.

### RNA extraction, library construction, and illumina sequencing

The total RNA was extracted from the RH and Root-RH samples using Zymo Direct-Zol RNA isolation kit (Zymo Research, Irvine, CA, United States) as per the prescribed protocol. The isolated RNA samples were then analyzed using NanoDrop for their quantity and quality checks. To assess their integrity, samples were run on 1%TBE gel, and the RNA integrity (RIN) value was estimated using Bioanalyzer 2100 RNA pico chip as per the manufacturer’s instructions (Agilent Technologies, Palo Alto, CA, United States). The intact RNA samples with a RIN value of more than 7.9 (range: 7.9–9.5) were proceeded with the Illumina sequencing protocol (Supplementary Figure 1D). The libraries were generated using TruSeq Stranded Total RNA Library Prep Plant kit with plant rRNA depletion (Illumina Inc., United States) according to the manufacturer’s protocol. The libraries were sequenced to generate 150 bp paired-end reads using Illumina Hiseq2500 according to the manufacturer’s instructions. The Illumina reads generated from all the samples were submitted to the Sequence Read Archive (SRA) at the National Center for Biotechnology Information (http://www.ncbi.nlm.nih.gov/sra; BioProject ID: PRJNA857918).

### Reads processing and expression profiling

The Illumina sequenced reads were demultiplexed and assessed for their quality. The raw reads generated from the eight samples (LPRH1 and 2; NPRH 1 and 2; LPRT 1 and 2; NPRT 1 and 2; LPRH, low phosphorus root hair; LPRT, low phosphorus Root-RH; NPRH, normal phosphorus root hair; and NPRT, normal phosphorus Root-RH) were subjected to a quality check of reads using FastQC (version 0.11.9; Andrews, 2010). Reads were subjected to quality trimming and adaptor removal using the TrimmomaticV0.32 tool (Bolger et al., 2014). High-quality Illumina reads were pseudo-aligned to the indexed chickpea reference transcriptome, and the read counts of transcripts were estimated using the Kallisto program (Bray et al., 2016). The summary of pseudo alignment for all the samples is summarized in Supplementary Table 1.

Read counts of the transcripts were imported and transformed to unigene counts (of protein-coding genes) through the txiimport() (Soneson et al., 2015) function using “lengthscaledTPM.” Lowly expressed unigenes were removed using a cutoff of counts per million (CPM) > 1. Further, counts were normalized using the calcNormFactors() function in edgeR (Robinson et al., 2010) using the “TMM” method. For each comparison, results were plotted as log_2_CPM (Supplementary Figures 2A–D). Filtered and normalized counts were subjected to differential gene expression analysis using the limma-voom pipeline (Ritchie et al., 2015). The dataset was then used to calculate log_2_FC, *p* values, and adjusted *p* values. Principle component analysis (PCA) was performed using the prcomp() function, and a PCA plot was made using ggplot2. Further, all the comparisons were compared using an upset plot.

### Functional annotation, identification of GO terms, and enrichment analysis

Gene ontology (GO) terms for *Cicer arietinum* were assigned using Blast2GO (Gotz et al., 2008). The proteome of chickpea was blasted with Arabidopsis and Medicago proteome using NCBI blastp (Johnson et al., 2008) to identify the closest homologs. Further, mapping and annotation were performed for assigning GO terms for the chickpea proteins. Enrichment analysis of the set of genes was performed using the enricher() function in the clusterProfiler package (Wu et al., 2021) with a *q*-value cutoff <0.1. The GO enrichment analysis results were visualized as various plots using the enrichplot package (Wu et al., 2021). Transcriptional factors and regulators were identified using the iTAK online (v1.6) database (Zheng et al., 2016).

### Promoter analysis

Promoter sequences were retrieved for the chickpea genes using GFF and reference genome fasta files from RefSeq (O’Leary et al., 2016). The required subset was used to perform the identification of root hair elements (RHEs) in the promoter sequences using FIMO software (Grant et al., 2011) of MEME suite v5.4.1 (Bailey et al., 2015). A stringent cutoff of value of *p* = 10^−4^ was used to identify the significant hit.

### Validation of RNA-seq by qPCR

Real-time quantitative PCR (qPCR) was performed to validate selected genes in ICC4958 for their tissue specificity and preferentiality. Primers were designed using NCBI’s Primer-BLAST and are summarized in Supplementary Table 2. One microgram of RNA was used to synthesize cDNA using Applied Biosystems™ High-Capacity cDNA Reverse Transcription Kit. Synthesized cDNA was then diluted to 1:5 with ddH_2_O before using as a template for qPCR. The reactions were performed using Applied Biosystems 7500 Real-Time PCR System with SYBR Green chemistry (Applied Biosystems, United States) with three technical and two biological replicates. The relative expression (2^−ΔΔCt^) of each gene was calculated with respect to the housekeeping gene eukaryotic elongation factor (CaEef1a) using a modified Livak method (Livak and Schmittgen, 2001).

### Statistical analyses

All the statistical analyses were undertaken using R version 4.1.2.^1^ The graphs other than specifically mentioned were generated using the ggplot2 package.

## Results

### Root architecture and root hair response of chickpea to low phosphorus availability

**Figure 1:**
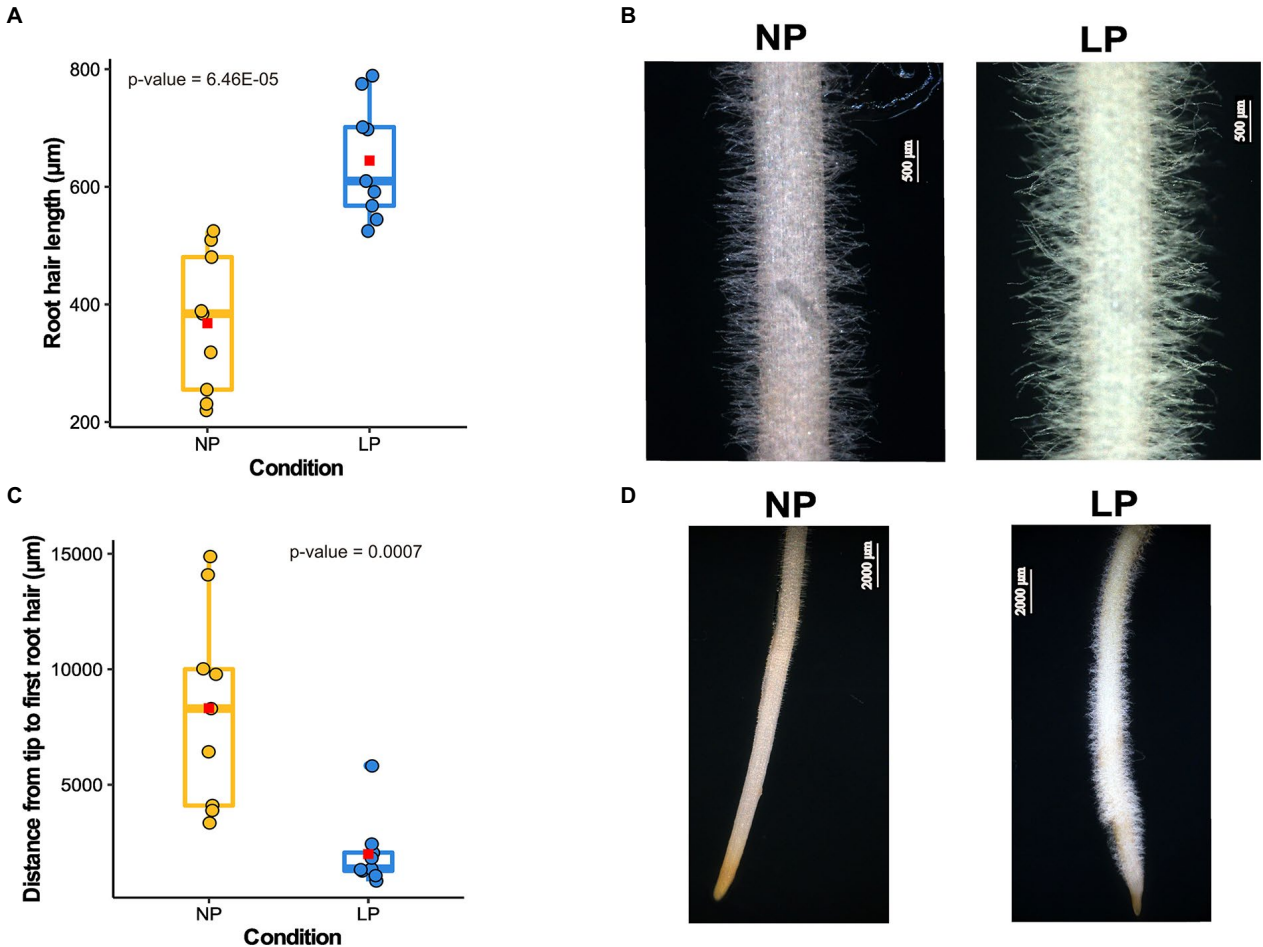
Root hairs (RH) growth response to low phosphorus (LP) in chickpea. **(A)** Box plot depicts the difference in RH length in normal phosphorus (NP) and LP conditions (*n* = 9). The *x*-axis represents the RH length, and the *y*-axis denotes different conditions. The average RH length for each condition is represented on the box plot as a red square, and value of *p* shows the significant difference calculated using a two-tailed *t*-test. **(B)** The microscopic images of the mature zone of chickpea root depicting the visual difference in RH length in NP and LP conditions. **(C)** Box plot displays the difference in the distance from the first visible RH to the root tip (*n* = 9). In this plot, the *x*-axis represents the distance from the root tip to the first visible RH, and the *y*-axis denotes different conditions. The average distance for each condition is designated as a red square, and the value of *p* shows the significant difference calculated using a two-tailed *t*-test. **(D)** Images of chickpea root in NP and LP conditions show the ectopic RH in LP near the root tip, resulting in a shorter distance from the root tip to the first visible RH.

Chickpea, like other crops, modulates its root architecture upon low Pi availability (Figure 1; Supplementary Figure 3). Seedlings grown under LP conditions exhibited a shorter primary root (Supplementary Figure 3A) and an increased lateral root density and average root diameter compared to those under NP conditions (Supplementary Figures 3C,D). The average RH length observed under NP conditions was 368 μm, whereas, under LP conditions, it was increased to an average length of 644.65 μm (Figures 1A,B). Also, the distance between the emergence of the first visible RH from the root tip is significantly increased in the case of NP (3.3 mm) compared to 1.99 mm under LP (Figures 1C,D). A shorter distance from the root tip to the first visible RH in LP results from the production of ectopic RH near the tip. An increase in the RH length and lateral roots density in LP conditions can facilitate more Pi absorption.

### Summary of RNA-sequencing results

**Figure 2:**
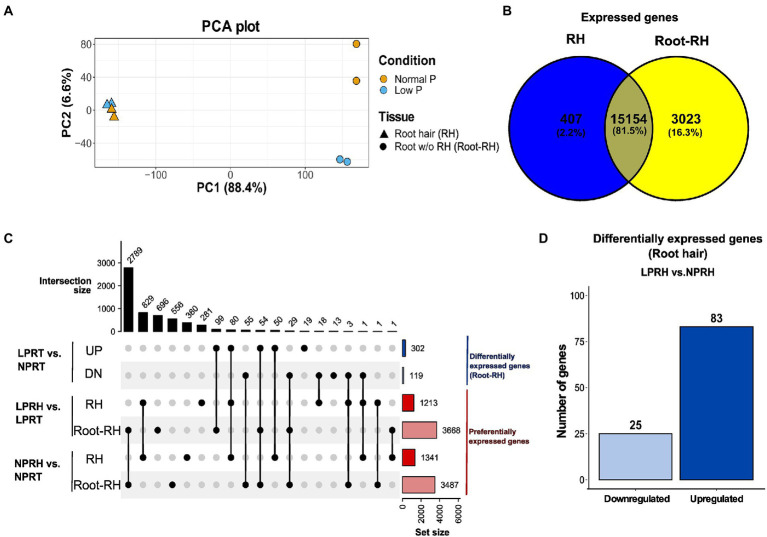
Summary of the RNA-seq results in root hairs (RH) and root without RH (Root-RH). **(A)** Principal component analysis (PCA) plot (PC1 vs. PC2) of all the samples in normal phosphorus (NP) and low phosphorus (LP) conditions. Triangle represents RH samples and the circle Root-RH samples. The yellow in PCA denotes NP, and the blue denotes LP conditions. Variation explained by each principal component is denoted in brackets. **(B)** Venn Diagram represents the comparison between the expressed active genes in RH (blue) and Root-RH (yellow) tissue in both conditions. **(C)** The comparisons are analyzed using an upset plot, denoting the intersection size on the upper left and set size on the lower right. Blue-colored sets represent differentially expressed genes in Root-RH between NP and LP conditions, and red-colored represent preferentially expressed genes in RH compared to Root-RH in each of the two conditions. **(D)** Bar graph showing differentially expressed genes in RH between NP and LP conditions. LPRH, low phosphorus root hair; LPRT, low phosphorus Root-RH; NPRH, normal phosphorus root hair; NPRT, normal phosphorus Root-RH; UP, upregulated; and DN, downregulated.

A total of 302.7 M fragments were sequenced using paired-end Illumina sequencing with an average GC content of 43.8% and a Q30 value of 92.38%. The reads were pseudoaligned to the chickpea reference transcriptome using Kallisto with an average alignment percentage of 77.54% (Supplementary Table 1). Libraries were subjected to principal component analysis (PCA) using the read counts. In PCA, PC1 explained 88.4% variation, corresponding to the difference between tissue samples (RH and Root-RH), and PC2 explained 6.6% variation, corresponding to the difference between conditions (NP and LP; Figure 2A). Root-RH had higher transcriptome modulation under LP compared to RH. Surprisingly, only a slight difference was observed between RH samples under LP and NP conditions. We performed two kinds of analysis using the dataset: (i) differential expression analysis between LP and NP conditions of both the tissue samples, RH (LPRH vs. NPRH) and Root-RH (LPRT vs. NPRT), and (ii) preferential expression analysis between RH and Root-RH for each of the conditions, LP (LPRH vs. LPRT) and NP (NPRH vs. NPRT; Figure 2C).

### Differentially expressed genes between low and normal phosphorus conditions

We performed differential expression analysis in RH and Root-RH tissues to identify LP responsive genes. As discussed, higher variation between LP and NP was observed in Root-RH samples. Therefore, the cutoff for Root-RH was considered |log2FC| ≥ 2 at adjusted value of *p* ≤ 0.1 compared to a less stringent cutoff, |log_2_FC| ≥ 1 at adjusted value of *p* ≤ 0.3 for RH (Supplementary Figures 4A,B). The number of differentially expressed genes identified in RH (108) with a less stringent cutoff was relatively lower than those identified in Root-RH (421). Thus, LP resulted in subtle transcriptomic changes in elongated (mature) RH compared to Root-RH. This suggests that LP responsive cellular signaling is more pronounced in the early phase of RH development.

#### Differentially expressed genes between low and normal phosphorus conditions in RH

**Figure 3:**
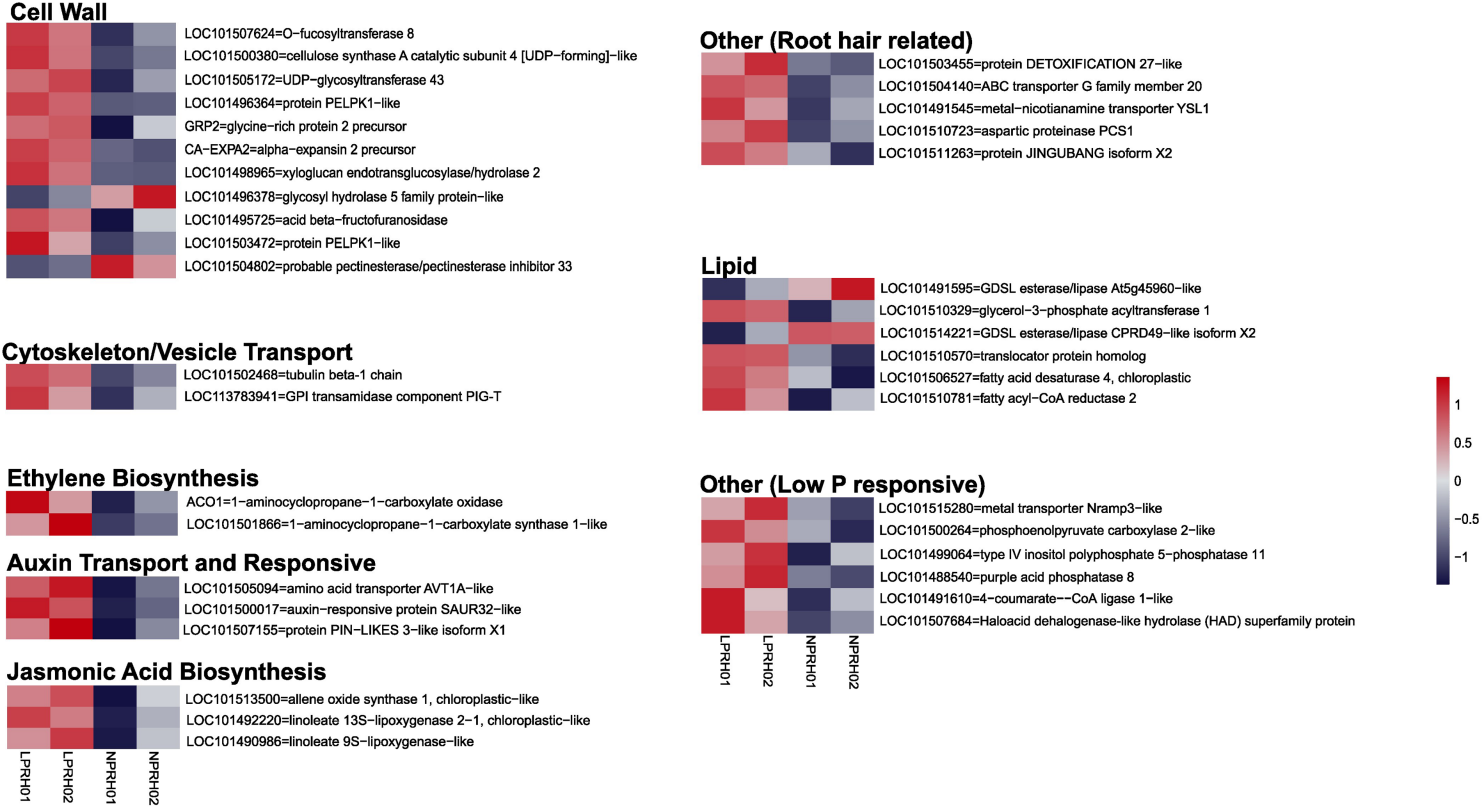
Categorization of differentially expressed genes (DEGs) between normal (NP) and low (LP) phosphorus conditions in root hairs (RH). Heatmaps depict differentially expression of genes using row scaled log_2_ counts per million (CPM) values, and DEGs are denoted using the gene id and description. DEGs are categorized according to their function and role in RH development or LP response. Each category can contain both up- and downregulated genes.

In mature RH, 83 genes were upregulated, while 25 were downregulated in LP conditions (Figure 2D; Supplementary Table 3). A subset of these genes which have known functionality in RH cell development or LP response were classified according to their known biological functions, namely cell wall synthesis and remodeling, cytoskeleton/vesicle transport, auxin response and transport, ethylene and jasmonic acid (JA) biosynthesis, lipid synthesis and remodeling, and LP responses (Figure 3).

Most upregulated genes were related to RH cell development. The cell wall remodeling genes like *expansin (CaEXPA2)* and *xyloglucan endotransglucosylase/hydrolase (XTH)* had higher expression depicting an increase in cell wall loosening in RH during LP. Also, cell wall synthesis genes like *cellulose synthase A catalytic subunit 4* and *O-fucosyltransferase 8* were upregulated (Figure 3). In RH under LP conditions, cytoskeleton/vesicle transport-related genes like *tubulin-beta1 chain* and *GPI transamidase component PIG-T* were upregulated (Figure 3). Induction of these processes in mature RH corresponds to enhanced RH tip growth during LP.

Root hair development processes are under the control of phytohormonal regulations predominantly, JA, ethylene, and auxins. In RH, ethylene (*ACO1* and *ACC synthase*) and jasmonic acid (*AOS* and *LOXs*) biosynthetic genes depicted higher expression in LP. Also, auxin transport and responsive genes were upregulated, showing auxin transport and signaling modulation during LP in RH (Figure 3). Thus, in mature RH, genes involved in RH development and phytohormonal biosynthetic, transportation, and signaling processes together orchestrate the increase in RH length during LP.

Apart from RH development, low P responses were also activated. The remodeling of lipid membranes primarily enhances the efficiency of P utilization. In RH under LP, we observed differential expression of various lipid-related genes like *CaGPAT1*, *CaFAD*, translocator protein, and *fatty acyl CoA reductase* were upregulated; however, GDSL esterase/Lipases were downregulated in LP (Figure 3). Further, genes that either facilitate P uptake or metabolic changes during LP were upregulated, like metal transporter, phosphatases, HAD domain-containing protein, and PEP carboxylase (Figure 3).

#### Differentially expressed genes between low and normal phosphorus conditions in root-RH

Dicots modify their root architecture in LP conditions to enhance the explorative area for P acquisition (Niu et al., 2013). The significant changes in root architecture include increased RH length, lateral root density, and shortening of the primary root. This plasticity is facilitated through significant transcriptomic modulation, which was also observed in chickpea roots under LP treatment (Supplementary Figure 4B). A total of 302 genes were upregulated, and 119 were downregulated in Root-RH under LP (Figure 2C).

**Figure 4:**
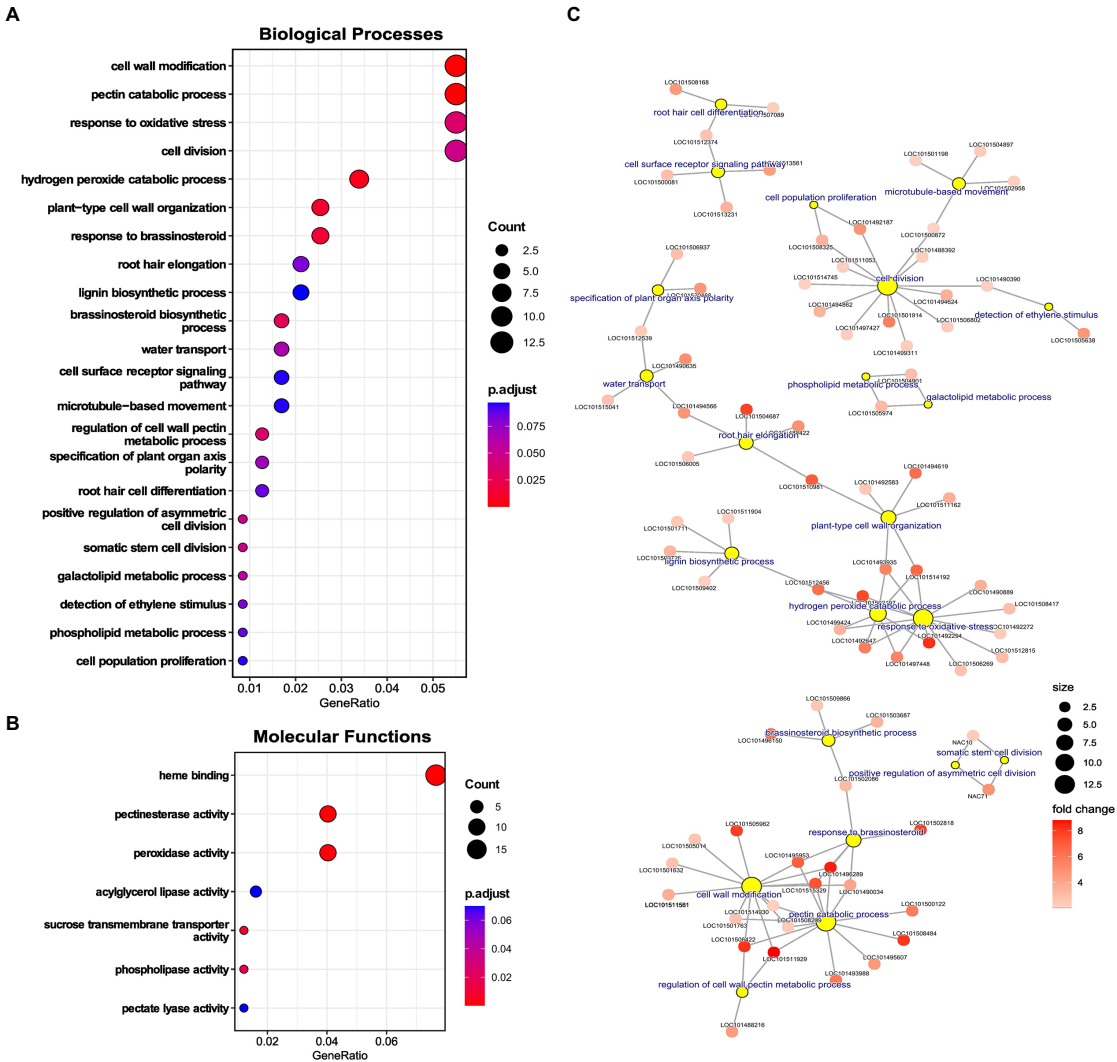
Enrichment analysis of upregulated genes in low phosphorus (LP) conditions in Root-RH. **(A)** Dot plot of enriched gene ontology (GO) terms for biological processes (BP) and **(B)** Molecular functions (MFs). The dot size represents the count of the genes under the term, and the color represents the adjusted value of *p* of enrichment. **(C)** Network of selected BP terms displaying genes under each term. The yellow circle represents the central nodes as BP term, and the sub-nodes are genes included in that term. The size of the yellow circle represents the gene count, and the color gradient of the sub-nodes (genes) depicts the fold change of the upregulated gene.

A complete gene set of upregulated genes was used to perform GO enrichment and identify significantly enriched processes. Various terms associated with cell wall were enriched: for biological processes (BPs), namely “cell wall modification,” “pectin catabolic processes,” “plant-type cell wall organization,” “lignin biosynthetic process,” and “regulation of cell wall pectin metabolic process,” and molecular functions (MFs) including “pectinesterase activity” and “pectate lyase activity” (Figures 4A,B). Genes associated with cell wall-related terms were majorly cell wall loosening proteins, like extensins, peroxidases, XTHs, pectate lyases, and pectin methyl esterases (Figure 4C). Further, genes for lignin biosynthesis and Casparian strip membrane proteins were also upregulated, depicting alteration of apoplastic barrier in chickpea roots under LP (Figure 4C).

Terms related to oxidative stress like “response to oxidative stress” and “hydrogen peroxide catabolic process” were also enriched (Figure 4A). Prominently, genes like peroxidase were included in these processes (Figure 4C). Various cell division and differentiation-related terms were also enriched, including “cell division,” “somatic cell division,” “asymmetric cell division,” “cell population proliferation,” and “specification of plant organ axis polarity.” Among phytohormonal processes, “response to brassinosteroid” and “brassinosteroid biosynthetic process” were enriched (Figure 4A).

Apart from specific processes, terms related to RH development like “root hair elongation” and “root hair cell differentiation” were significantly enriched (Figure 4A). Genes associated with “root hair elongation” were aquaporin PIP2-5-like, non-classical arabinogalactan protein 30, PGR5-like protein 1A, and bHLH85-like (Figure 4C). The term “root hair cell differentiation” had three associated genes, adenine nucleotide alpha hydrolases-like, probable inactive receptor-like protein kinase, and glycerophosphodiester phosphodiesterase GDPDL3 (Figure 4C). Besides the enriched terms, RH initiation genes like *ROP guanine nucleotide exchange factor (ROPGEF)* were also upregulated (Supplementary Table 4). Upregulation of RH genes in Root-RH samples in LP can be instrumental in RH differentiation and initiation and can also convey induced RH growth signaling (through RSL TFs) to mature RH, as observed in the RH dataset. Therefore, these genes might be involved in producing ectopic RH near the RH tip as a response to LP.

Among the enriched processes, terms associated with the P starvation response of lipid remodeling were also enriched. For BPs, terms like “phospholipid metabolic process” and “galactolipid metabolic process” and for MFs, “phospholipase activity” and “acyglycerol lipase activity” were highly enriched.

Processes and functions enriched in the upregulated set were not enriched in downregulated one. For downregulated genes, enriched BPs included “meristem determinacy,” “meristem initiation,” “coumarin biosynthesis process,” “response to nitrate,” “carbohydrate transporter activity,” “negative regulation of defense response to insect,” “defense response to insect,” and “regulation of jasmonic acid-mediated signaling pathway” (Supplementary Figures 5A,C). For MFs, phytohormonal-related functions like “jasmonic acid hydrolase,” and “gibberellin 20-oxidase activity” were enriched. Besides these, “phosphatidyl ethanolamine binding,” “sugar transmembrane transport,” and “thioredoxin-disulfide reductase activity” were also enriched (Supplementary Figure 5B).

### Preferentially expressed genes in RH and root-RH under low and normal phosphorus conditions

Root-RH and RH displayed distinctiveness in their transcriptomes. In Root-RH, 18,177 unigenes were expressed compared to 15,561 in RH combined in both LP and NP conditions (Figure 2B). Of the expressed active genes, 407 were specific to RH, while 3,023 were specific to Root-RH against each other (Figure 2B). Further, we analyzed the preferential genes in RH and Root-RH in LP and NP conditions, and for each comparison, a cutoff of |log_2_FC| ≥ 2 at an adjusted value of *p* ≤ 0.1 was considered (Supplementary Figures 4C,D). We identified that in LP, 1,213 genes were preferentially expressed in RH and 3,668 in Root-RH (Supplementary Table 5). In NP, 1,341 genes were preferential for RH, and 3,487 were preferentially expressed in Root-RH (Figure 2C; Supplementary Table 6). Here, we discussed preferential genes of RH in LP and NP conditions and a set of preferential genes specific to LP.

#### Preferential genes expressed in RH

**Figure 5:**
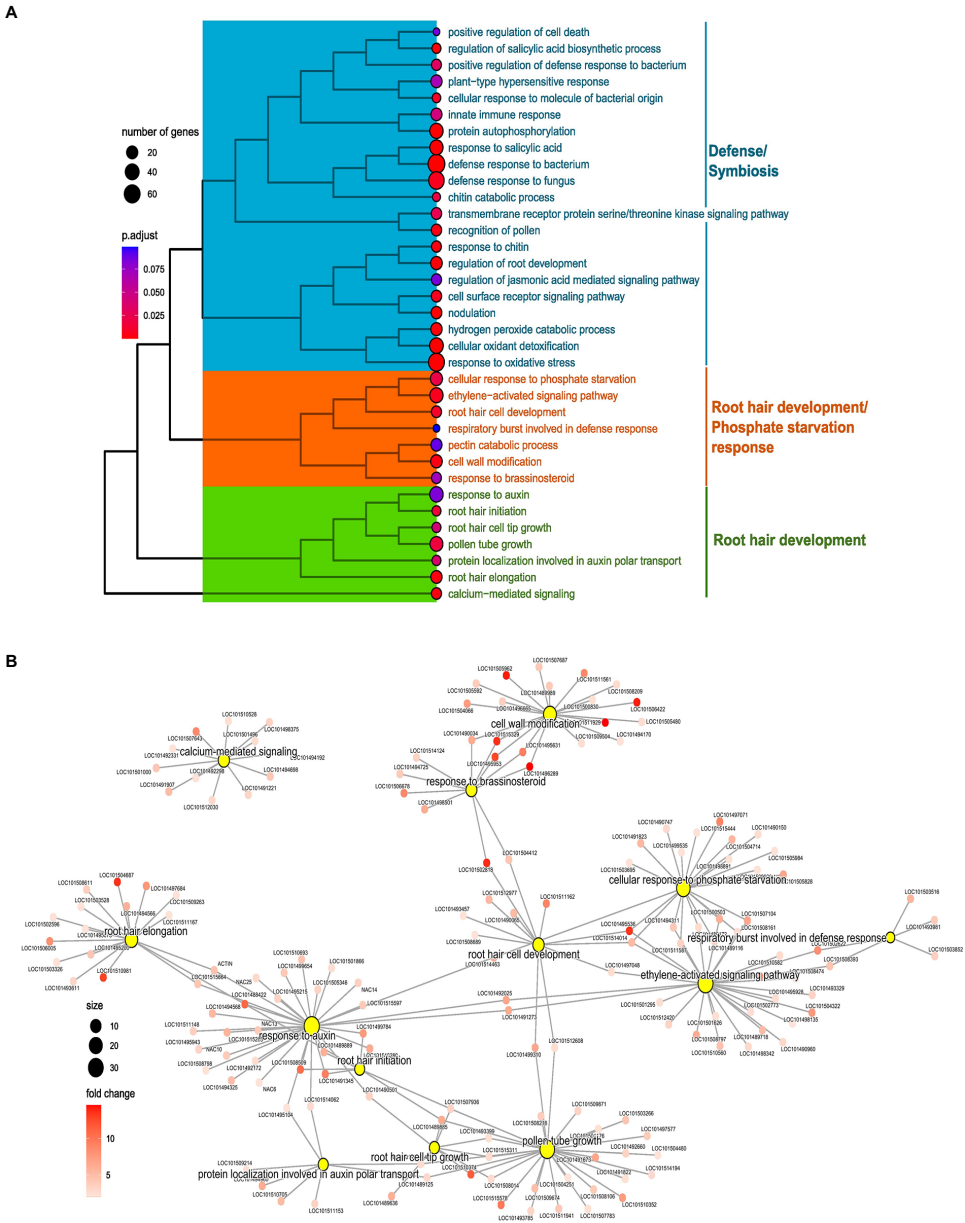
Enrichment analysis of preferentially expressed genes in root hairs (RH) in normal phosphorus (NP, NPRH vs. NPRT). **(A)** Tree plot representing the clustering of enriched terms of biological processes (BPs) for preferentially expressed genes in RH in NP. The terminal branch of the tree represents an enriched term, and the size of the dot depicts the number of genes assigned from the preferential gene set to that term. The color represents the adjusted value of *p* of the enrichment. The tree plot is clustered and categorized into three clusters. The functionality that each cluster represents is denoted on the left-hand side. **(B)** Network of BP terms (central nodes) and the preferentially expressed genes (sub-nodes) from the orange and green clusters. The size of the central nodes represents gene count, and the color gradient of sub-nodes depicts fold change of preferentially expressed genes.

Root hair preferential genes were subjected to GO enrichment for BPs, identifying various enriched processes. These BPs were largely clustered into three clusters (C1–3) depending upon common genes that each term carries. The three clusters were annotated as per the terms, namely C1, Defense/Symbiosis; C2, and Root hair development/Phosphate Starvation response; and C3, Root hair development. Regulatory terms and phytohormonal-related terms were present in each cluster as per their similarity with the remaining terms (Figure 5A). Preferential analysis was performed separately for LP and NP; however, results in this section are discussed combined for both the conditions (Figures 5A,B).

In C1, microbial interaction-related terms were present in LP and NP conditions. Many are associated with recognition and response to molecules of bacterial and fungal origin. Further, various enriched processes were clearly related to symbioses like nodulation, negative regulation of immune response, and negative regulation of defense response to oomycetes. Apart from symbiotic processes, processes for defense against fungal and bacterial microbes were also enriched (Figure 5A; Supplementary Figures 6A,B).

The term “cellular response to phosphate starvation” was enriched in LP and NP conditions. Genes included in this term were *phosphoenolpyruvate carboxykinase* (ATP; LOC101503695), phosphate transporter *PHO1 homolog 9*, *MYB108*, *ribonuclease-1* & *3*, *inorganic phosphate transporter 1-4-like* (LOC101515444), *probable inorganic phosphate transporter 1–3* (LOC101497071), *probable inorganic phosphate transporter 1–9* (LOC101504714), *probable inorganic phosphate transporter 1–3* (LOC101490150), *SEC12-like protein 1* (LOC101505984), *O-acyltransferase WSD1-like* (LOC101509936), and *phospholipase D zeta 1-like* (LOC101514463; Figures 5A,B). Preferential expression of these proteins, particularly of various P transporters in RH, shows the importance of RH in the uptake of P from the soil. Further, many WRKY TFs were common between ‘cellular response to phosphate starvation and “activation of ethylene signaling,” hinting toward the role of ethylene in orchestrating LP response in RH through WRKY (Figure 5B).

In C2 and C3, processes related to RH development and phytohormonal control were predominantly present. Processes directly linked to RH development were “root hair initiation,” “root hair elongation,” “root hair cell tip growth,” and “root hair cell development” (Figure 5A). These terms contain genes with diverse biological functions vital for RH development and its regulation. Apart from these, terms for various processes that play an essential role in RH development like “cell wall remodeling,” “calcium-mediated signaling,” and “respiratory burst involved in defense response” were also enriched (Figure 5A). Many genes from the RH development and accessory processes were common between phytohormonal terms like “ethylene activation signaling,” “response to auxin,” “response to brassinosteroid,” and “protein localization involved in auxin transport,” depicting the importance of these phytohormones for the RH development (Figures 5A,B).

#### Specific preferential genes in root hairs under low phosphorus conditions

**Figure 6:**
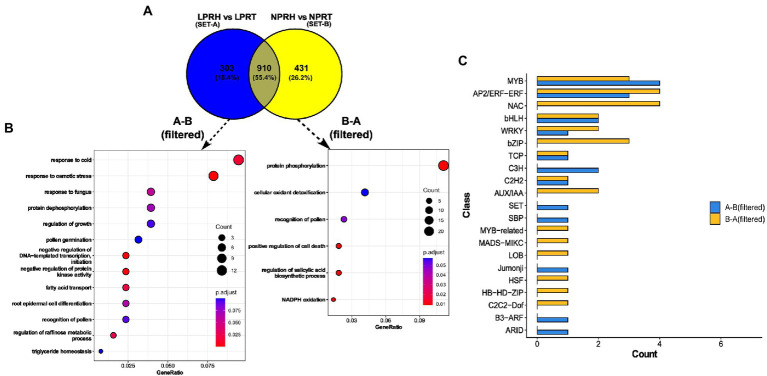
Preferentially expressed genes in root hairs (RH) specific to low phosphorus (LP) conditions. **(A)** Venn diagram comparing preferentially expressed genes in normal phosphorus (NP) (set A) and LP (set B) conditions. **(B)** Dot plot depicting enrichment of LP-specific (A-B, filtered) and NP-specific (B-A, filtered) genes. Here, the size of the dot represents gene count, and color depicts the adjusted value of *p* of enrichment. **(C)** Bar graph depicting the count of transcription factors (TFs) and regulators (TRs) in each class for LP (A-B, filtered) and NP (B-A, filtered) specific datasets.

We selected unique genes that were preferentially present in each of the conditions. Genes were filtered as per the difference (>1) between their preferential fold change values in LP and NP conditions. Out of 303 unique preferential genes in LP, 157 were left after filtering. Of 431 unique preferential genes in NP, 260 were left after filtering. Both the filtered sets were subjected to GO enrichment for BP, and the results were presented as dot plots (Figures 6A,B).

Enriched terms were different in both the sets except “recognition of pollen.” Preferential gene set specific to LP had enriched terms related to lipids like “fatty acid transport” and “triglyceride homeostasis.” Also, RH development terms like “pollen germination,” and “root epidermal cell differentiation” were enriched in LP. Interestingly, both “negative regulation of protein kinase activity” and “protein dephosphorylation” were enriched specifically in LP, and contrastingly, “protein phosphorylation” was enriched in NP. Also, cell death-related processes like “positive regulation of cell death” and “regulation of biosynthesis of salicylic acid” were present in the NP dataset. In summation, preferential genes specific to LP were related to RH development, LP response, and dephosphorylation. In contrast, cell death genes were enriched in mature RH in NP (Figures 6A,B). These observations hint toward the possible role of the longevity of RH in LP.

### Differentially regulated transcription factors and transcriptional regulators in various sets

We identified and compared TFs and TRs in two data sets—DEGs in Root-RH and preferential genes specific to LP and NP. Among upregulated genes in Root-RH under LP, 16 TFs and two TRs were identified. Transcription factors were distributed into 11 and transcription regulators into two classes (Supplementary Figure 7). For downregulated genes, 13 TFs distributed into six classes and one TR were identified. Transcription factors and regulators belonging to GNAT, HMG, GRAS, GARP-G2-Like, GRF, LOB, MADS-MIKC, TCP, and MYB were only present in the upregulated set. C2H2, ARID, and MYB-related were unique to downregulated TFs and TRs. A similar analysis was carried out for specific preferential genes: 16 TFs (nine classes) and three TRs (three classes) were identified for LP, and 24 TFs (14 classes) and two TRs (one class) for NP. TFs and TRs belonging to classes B3-ARF, ARID, Jumonji, SBP, SET, and C3H were unique to preferential gene set specific to LP. For NP, TFs and TRs belonging to classes, such as C2C2-Dof, HB-HD-ZIP, HSF, LOB, MADS-MIKC, MYB-related, AUX/IAA, bZIP, and NAC were unique and absent in LP (Figure 6C).

### Identification of root hair elements in promoters of exclusively and preferentially expressed genes in root hairs

Promoters’ sequences of RH exclusive genes were retrieved and analyzed for RHEs (5’WWMNTGNN(N)YGCACGWH3’) using FIMO (Kim et al., 2006). Of the 406 promoters, 129 had one or more RHEs (Supplementary Table 7). Similarly, for RH preferential genes in LP, of 1,210 promoters, 391 had one or more RHEs (Supplementary Table 8). A stringent cutoff of value of *p* = 10^−4^ was used for the promoter analysis.

### Validation of RNA-seq data through qPCR

Two sets of genes, four RH-specific genes (LOC101508484, LOC101504687, LOC101500876, and LOC101514257) and three Root-RH specific (LOC101511407, LOC101490071, and LOC101502234) were selected according to their expression values from RNA-seq. qPCR was performed for these selected genes, and the results showed a similar expression pattern in qPCR and RNA-seq, validating the RNA-seq results and the tissue-specificity of the samples (Supplementary Figure 8).

## Discussion

Root hairs are single-celled epidermal projections that play crucial role in facilitating mineral and water uptake. In soil mineral nutrient deficiencies, particularly P, RHs become longer and denser to facilitate higher P acquisition (Bates and Lynch, 1996; Figure 1). Thus, we performed tissue-specific transcriptome analysis to elucidate the developmental regulations in chickpea RH during LP. For this, tissue-wise and treatment-wise comparisons were made, including RH transcriptome between LP and NP conditions, Root-RH transcriptome between LP and NP, and among each other. Structurally, RH is much simpler than complex roots containing varied cell lineages. This difference in complexity is reflected in their transcriptome profile. Out of total expressed genes in both the RH and Root-RH tissues, RH of chickpea expressed only 2.2% of genes exclusively, compared to 16.3% in Root-RH. This result is quite similar to that observed in maize (3%; Hey et al., 2017) and in Arabidopsis (4%; Lan et al., 2013). Further, homologs of various known RH markers like expansin A-7 (LOC101504687) are preferentially expressed in RH with log_2_FC of 13.62 and are validated using qPCR (Supplementary Tables 5, 6; Supplementary Figure 8). These observations strengthen the accuracy of the RH isolation protocol and sample specificity.

### Tissue-specific transcriptomic changes in orchestrating root hair development in LP

Among the DEGs identified in Root-RH under LP, a part is related to RH development and initiation, as epidermal cells and bulges belong to Root-RH, not mature RH. In Arabidopsis, LP induces *RSL4* before or at the point of RH initiation, leading to accelerated RH tip growth in later stages (Datta et al., 2015; Vijayakumar et al., 2016). Similarly, in chickpea, a bHLH TF (LOC101488422) having 85% identity with RSL4 is upregulated in LP in Root-RH. Apart from RSL4, various genes that have known functions in RH initiation are also upregulated in chickpea Root-RH. Few RH cell differentiation genes like *GDPDL3* are also induced in LP. These changes in the transcriptome in Root-RH in LP could be involved in the induction of denser and ectopic RH.

The induction of RH development machinery upon LP is also observed in mature RH. In mature RH, processes related to tip growth like cell wall-related processes, cytoskeleton, vesicle transport, and lipid metabolism are induced in LP. Induction of RH tip growth is facilitated through phytohormones, most importantly ethylene, auxin, and jasmonic acid (JA). Interestingly, ethylene and JA biosynthesis and auxin-responsive and transport genes are induced in chickpea RH in LP. Further, among preferentially expressed genes specific to treatments, TFs AUX/IAA are expressed in NP and ARFs in LP, depicting suppression of auxin signaling in NP and activation in LP. Clearly, the RH response to LP is under the strict control of ethylene and auxin in chickpea, as observed in other crops and Arabidopsis (Rahman et al., 2002; Song et al., 2016; Bhosale et al., 2018).

### Low phosphorus response of chickpea roots besides induction of RH growth

Apart from an increase in RH growth, roots respond to LP through remobilizing phosphorus from Pi reserves, releasing exudates and acid phosphatases, and increasing lateral root number and density (Tjellström et al., 2008; Pérez-Torres et al., 2009; Bhadouria and Giri, 2021). Therefore, many genes that take part in LP tolerance are also upregulated in RH and Root-RH. Specially upon LP, lipid remodeling enzymes are induced like GDPD and FADs in RH (Rietz et al., 2010; Chen et al., 2011; Verma et al., 2021), and GPAT1, FAD4, fatty-acyl-CoA reductase and patatin like proteins in Root-RH (Rietz et al., 2010; Chen et al., 2011; Verma et al., 2021).

Recently, cell wall pectins are also considered a vital Pi reserve during LP in rice (Tao et al., 2022). For the mobilization of Pi from cell wall pectins, pectin methyl esterases (PMEs) are induced during LP (Tao et al., 2022). Similarly, in chickpea, pectin catabolic genes are upregulated in Root-RH in LP; these mainly include eight pectin esterase, three pectate lyase, and one polygalacturonase. All three classes of enzymes are involved in depolymerization and breakdown of cell wall pectin. Also, pectin catabolic genes are associated with the term “responsive to BR”; however, no such role of BR in the mobilization of Pi in LP is yet defined. Interestingly in rice, Pi mobilization from cell wall pectin is governed by JA and ABA (Fang Zhu et al., 2018; Tao et al., 2022). In the present dataset, genes for JA hydrolysis are downregulated in chickpea roots, hinting toward higher JA accumulation during LP (Supplementary Figure 5B). Further studies will be required to define the regulation of Pi mobilization from cell walls in chickpea and other legumes.

In chickpea, lateral root number and density increase during LP, and few transcriptomic changes in chickpea roots indicate this response. For example, genes involved in asymmetric and somatic cell division are also enriched in the upregulated dataset, and these processes are crucial for lateral root primordia formation (Schütz et al., 2021). Further, genes involved in the specification of polar organ axis polarity and water transport are also upregulated, and these processes are essential for the development and emergence of lateral roots (Péret et al., 2012; Reinhardt et al., 2016). Further, BR biosynthesis is enriched in the upregulated dataset, and BR-mediated auxin regulation is essential for LR formation in roots of Arabidopsis (Bao et al., 2004); upregulation of BR biosynthesis might have a similar role in chickpea. The transcriptomic modulations identified in chickpea roots can thus orchestrate various responses for providing tolerance to LP.

### Preferentially expressed genes in root hairs state development and functions of root hair

Preferentially expressed genes in chickpea RH are categorized into three categories according to their functional roles: RH development, Pi transport and responsiveness to phosphate, and microbial interaction.

The genes for RH development broadly consist of cell wall-related proteins, acyltransferases, aquaporins, RSL transcription factors, calcium signaling-related proteins, cytoskeleton proteins, and ROS metabolism and forming enzymes. These cellular functions and molecular processes are essential for RH initiation and tip growth (Campanoni and Blatt, 2007; Datta et al., 2011; Pires et al., 2013; Mangano et al., 2016; Salazar-Henao et al., 2016). Also, auxin transport genes are preferentially expressed for regulating RH development like Pattelins and Exocyst70A. Patellins are very well characterized in Arabidopsis for their involvement in the relocation of PIN1 for root gravitropism and shoot apical meristem development (Tejos et al., 2018). However, their functioning is not yet characterized for RH. Besides auxin, almost 30 genes are assigned under the term “ethylene activated signaling,” consisting of genes involved in ethylene signaling like ERFs, ERN2, RAP2-1, and WIN1 (ChunJuan and JinYuan, 2010; Müller and Munné-Bosch, 2015), and RH development like phospholipase D alpha-1 (Lin et al., 2020), extensin (Baumberger et al., 2001), and respiratory burst oxidase homolog H and E (Mangano et al., 2017). Interestingly, many of these genes are common with terms like “root hair cell development,” “response to auxin,” and “cellular response of phosphate starvation,” indicating the role of ethylene signaling in orchestrating RH developmental response in LP.

Lastly, a significant subset of preferentially expressed genes in RH belongs to terms associated with microbial interactions. Root hairs act as a harboring and entry site for various microbes involved in pathogenic and symbiotic associations (Ribaudo et al., 2006; Downie, 2010; Prieto et al., 2011; Pečenková et al., 2017). In leguminous plants, like chickpea, processes like attachment and attraction of rhizobia and infection thread formation involve RH and its molecular machinery (Esseling et al., 2003; Wheatley and Poole, 2018). Among RH preferential genes, 13 are associated with the term “nodulation” and are involved in initiating or establishing the nodule. Symbiotic bacteria guide the early response of the plant and infection thread formation through the sustained release of nod factors (Lhuissier et al., 2001; Esseling et al., 2003). The central regulator of nod factor transcriptional response is NSP1, and the receptor for Nod factor perception, the lysM domain receptor-like kinases like LYK2, are preferentially expressed in RH (Limpens et al., 2003; Smit et al., 2005). Also, many chitinases are highly expressed in RH, which are known to regulate nod-factor levels (Malolepszy et al., 2018). Moreover, major transcriptional regulators of nodulation, ERN1 and 2, are preferentially expressed in RH and together work in the root epidermis to establish rhizobia infection (Cerri et al., 2016). The subset also includes LIN1 ligase that interacts with VPY1 and VPY2, and this interaction controls endocytosis for establishing rhizobia infection and interaction with arbuscular mycorrhizal fungi (Liu et al., 2021). In the LP preferential dataset, terms like “negative regulation of defense response against oomycetes” are enriched, depicting the possible involvement of RH in the mycorrhizal association. In addition, at the transcription level, we identified the preferential expression of different receptor-like kinases and G-type lectin kinases (Sun et al., 2020). Besides symbiosis, these receptors are important effectors and recognizing sites for various pathogens and defense responses (Luo et al., 2017; Pham et al., 2020). Also, in the preferential RH dataset, many terms are enriched and associated with defense response, immune response, and hypersensitive response, depicting the involvement of RH molecular machinery in pathogen recognition and defense response. With such a diversified preferential gene repertoire in a single-celled RH, it is evident that RH performs diverse and crucial functions in the rhizosphere.

## Conclusion

**Figure 7:**
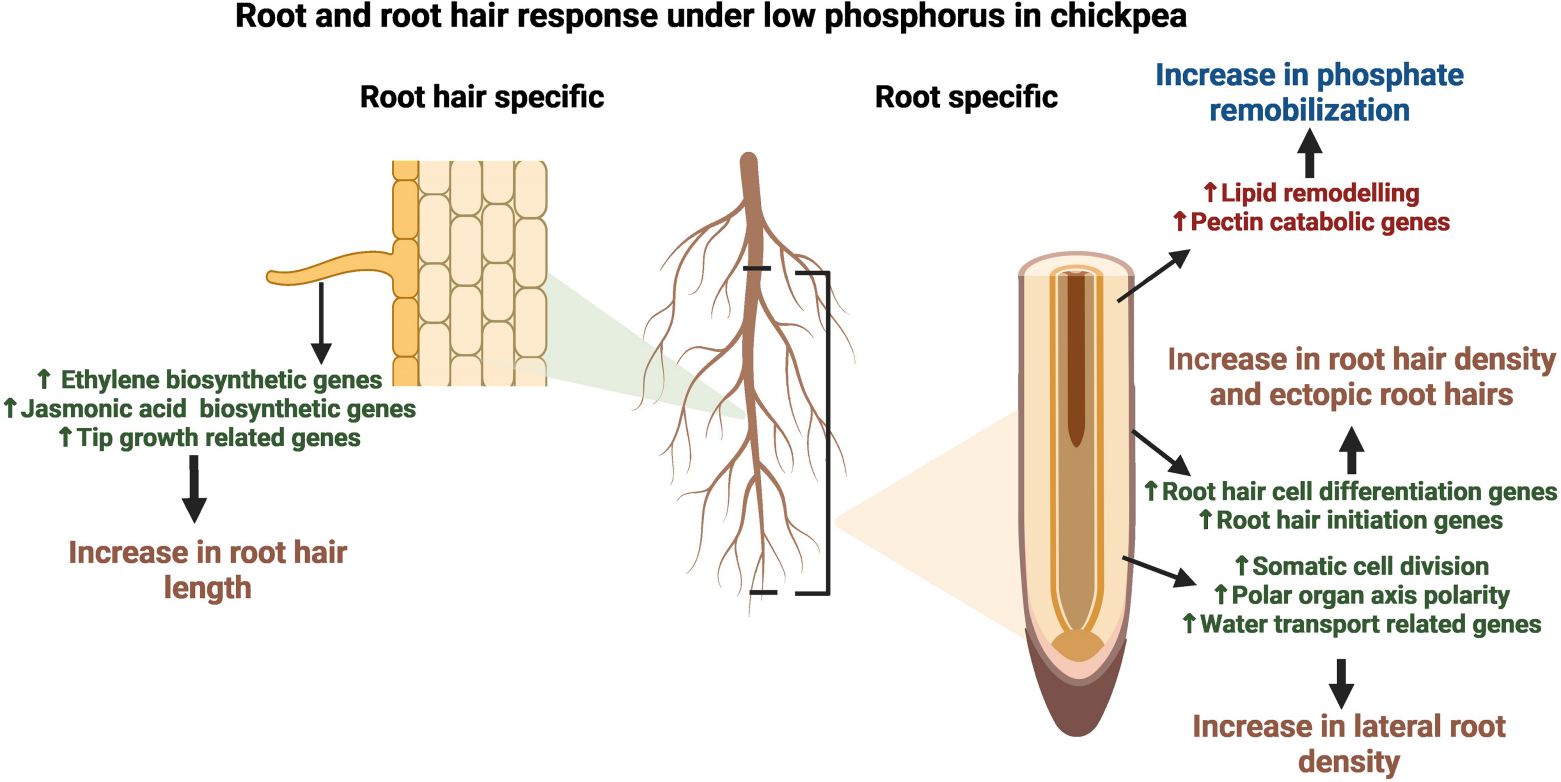
Summarized model of root and root hair (RH) response under low phosphorus (LP) in chickpea. The figure illustrates the tissue-specific upregulation of developmental and LP responses. The fully expanded RH depicts upregulation of ethylene and jasmonic acid biosynthetic genes, along with genes involved in tip growth processes like cell wall and lipid modulation and cytoskeleton/vesicle transport-related genes, which lead to an increase in root hair length under LP. In contrast, root without root hairs (Root-RH) depicted an increase in genes involved in RH cell differentiation and initiation, leading to an increase in root hair density and ectopic root hair formation. In addition, lateral root density increases in LP due to the upregulation of genes involved in processes like somatic cell division, polar organ axis polarity, and water transport. Further, both RH and root-RH showed upregulation of various LP tolerance genes; however, many pectin catabolism and lipid remodeling related genes are upregulated in root-RH for remobilization of phosphorus from the cell wall and lipids, respectively.

The present study aimed to determine the molecular changes in RH and Root-RH in response to LP and to elucidate the preferential transcriptome landscape of chickpea RH. Our transcriptomic and expression studies have revealed the marker genes like expansin A-7 and pectate lyase 2 for RH in chickpea, which adds to the existing catalog for various model plants, like Arabidopsis. The preferentiality of genes for RH clearly states the already known molecular functions like RH cell development and Pi starvation response but also depict the molecular players for emerging functional roles like microbial interactions (Figure 7). Further, upregulation of multiple chickpea genes in LP explained RH and root phenotypic plastic responses. For LP response of RH, transcriptomic changes start in the root itself for RH cell differentiation and then conveyed as upregulation of tip growth genes in elongated RH (Figure 7). Also, we elucidated tissue-specific molecular signatures for novel and already known tolerance mechanisms against LP. From this study, the identified TFs and essential genes could further be utilized to improve RH traits and develop LP tolerant chickpea varieties.

## Data availability statement

The data presented in the study are deposited in the NCBI repository, accession number PRJNA857918.

## Author contributions

PK and JG conceptualized the study. PK performed all the experiments, analyzed data, and wrote the manuscript. LP analyzed the data and edited the manuscript. BM performed qPCR experiment. JT edited the MS. JG supervised the study, edited the draft, and finalized the manuscript. All authors contributed to the article and approved the submitted version.

## Funding

Our research is funded by an institutional core grant and DBT-Innovative Young Biotechnologist Award (IYBA; BT/010/IYBA/2016/04).

## Conflict of interest

The authors declare that the research was conducted in the absence of any commercial or financial relationships that could be construed as a potential conflict of interest.

## Publisher’s note

All claims expressed in this article are solely those of the authors and do not necessarily represent those of their affiliated organizations, or those of the publisher, the editors and the reviewers. Any product that may be evaluated in this article, or claim that may be made by its manufacturer, is not guaranteed or endorsed by the publisher.

## Footnotes

^1^ www.r-project.org
