# Supplementary figures and images for: Root hair-specific transcriptome reveals response to low phosphorus in Cicer arietinum
Source: Front Plant Sci. 2022 Oct 4;13:983969. doi: 10.3389/fpls.2022.983969 (PMC9577374; doi:10.3389/fpls.2022.983969)

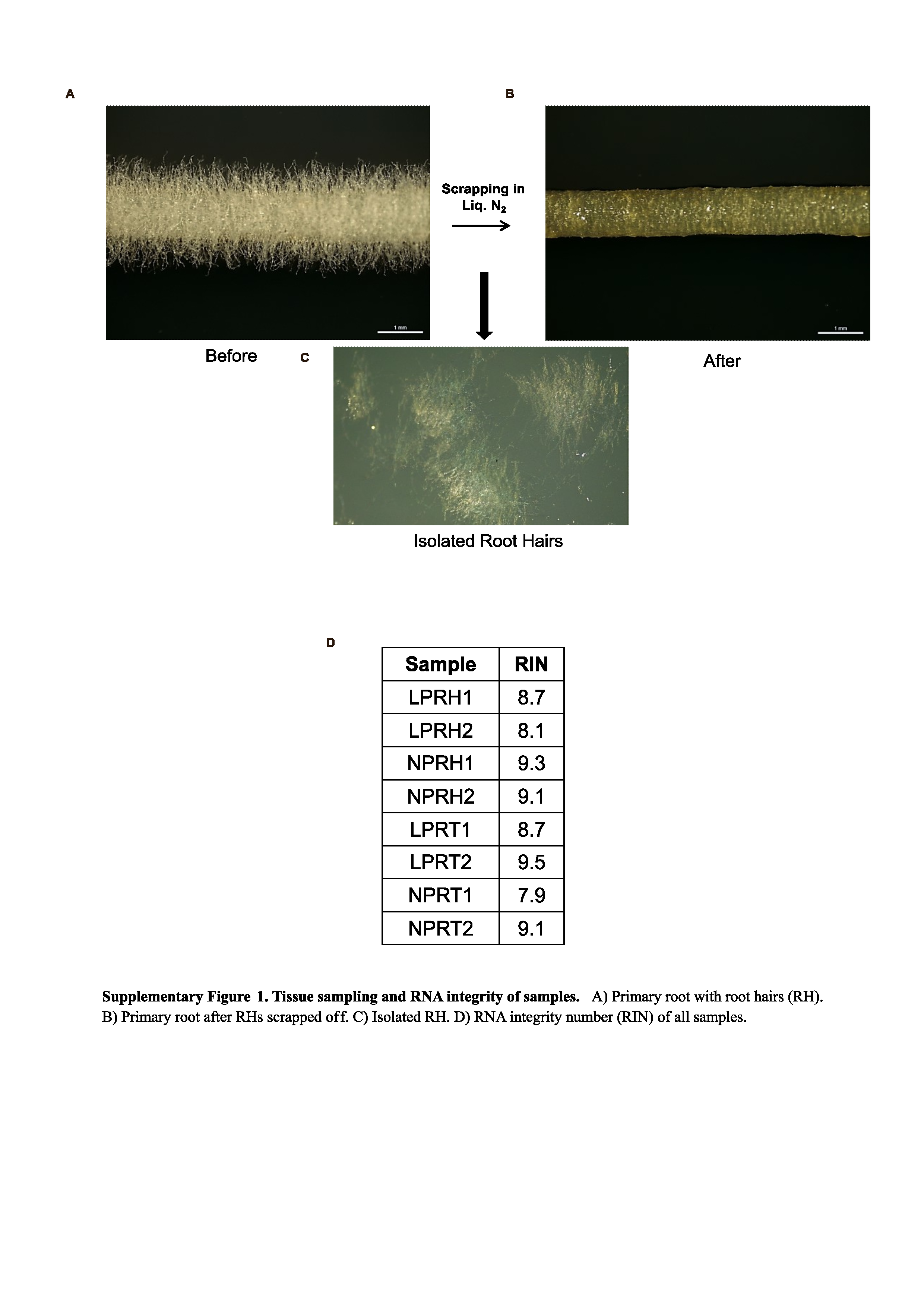

Supplement: Supplementary file 2 [file Image_1.TIFF]

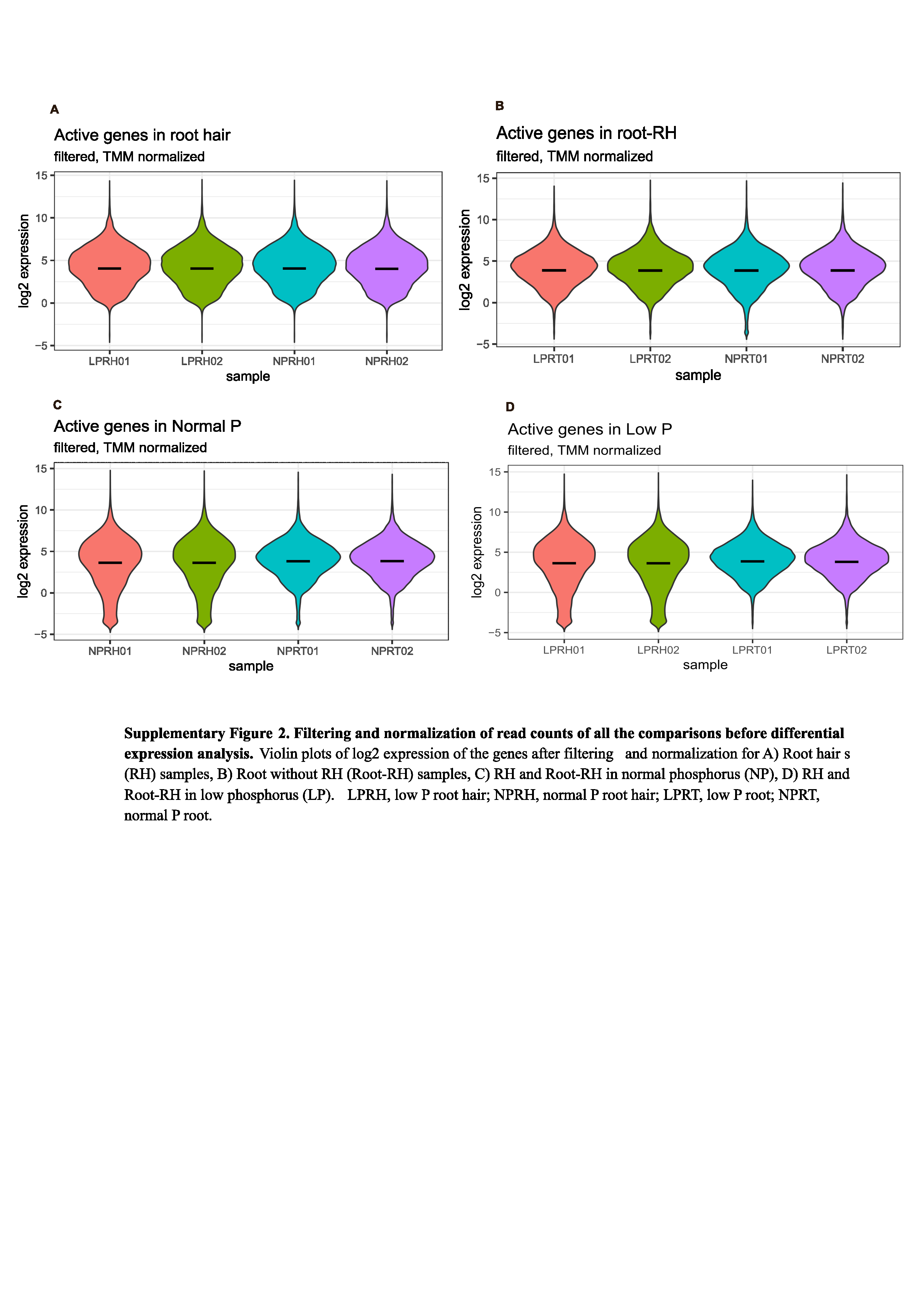

Supplement: Supplementary file 3 [file Image_2.TIFF]

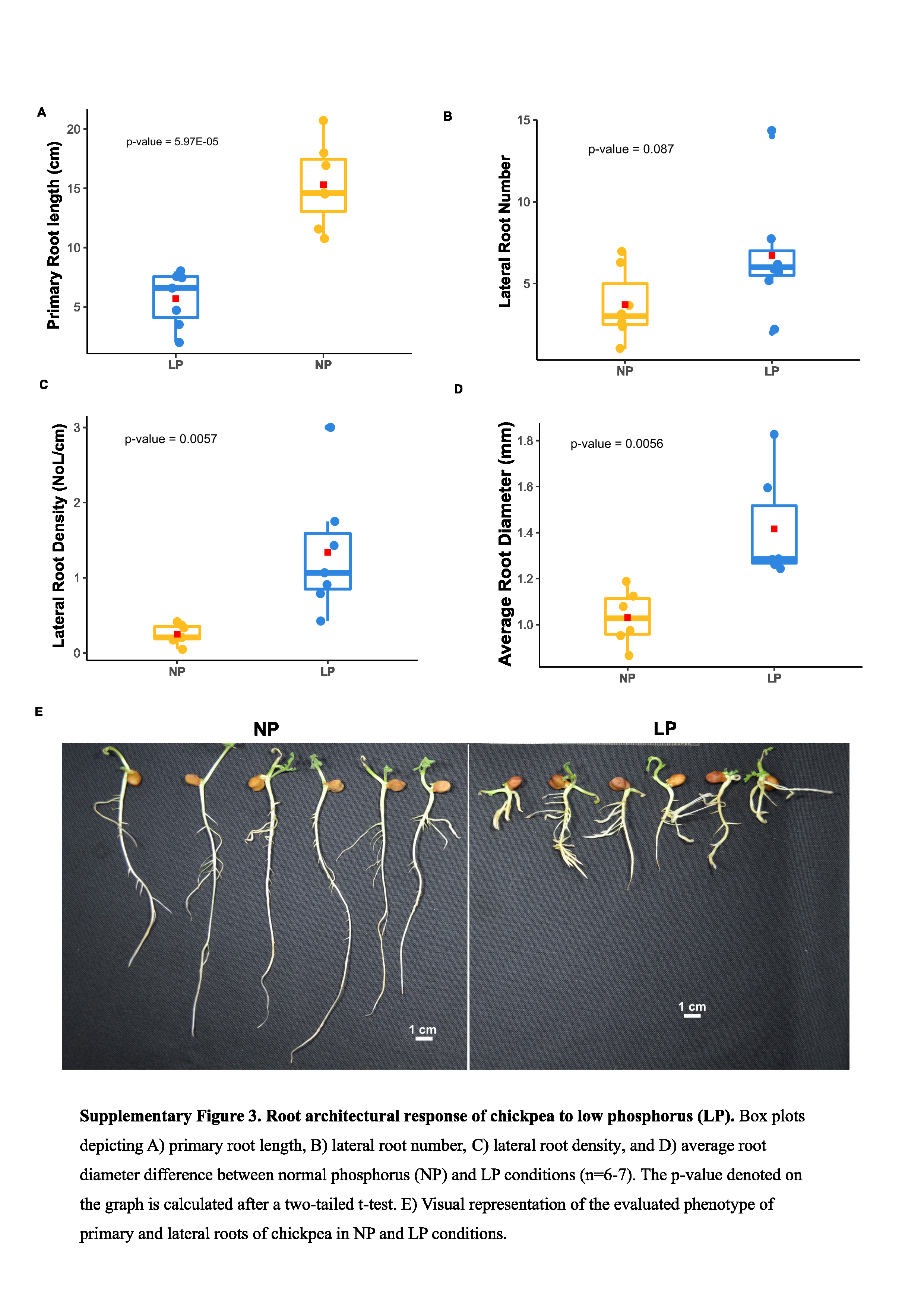

Supplement: Supplementary file 4 [file Image_3.TIFF]

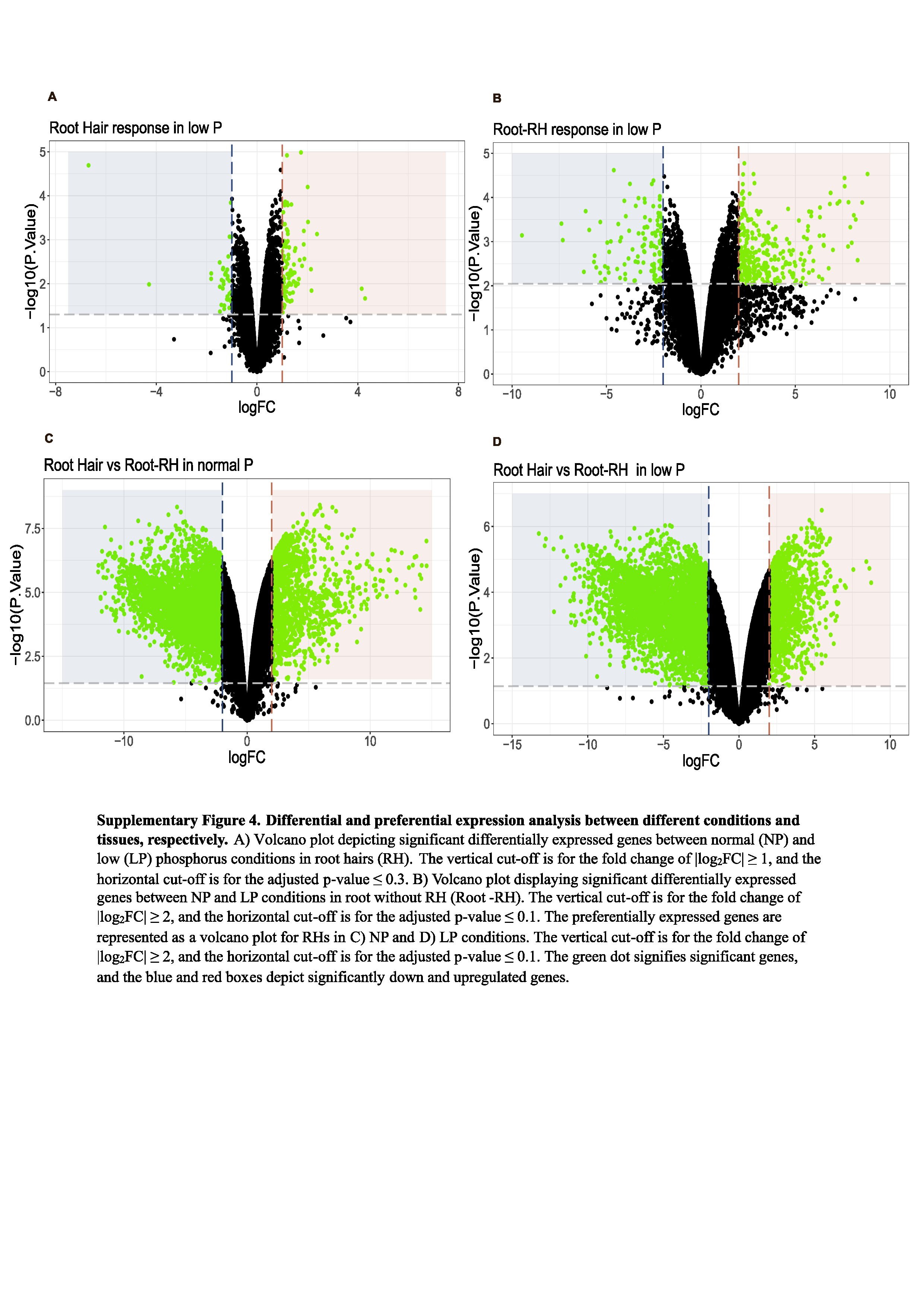

Supplement: Supplementary file 5 [file Image_4.TIFF]

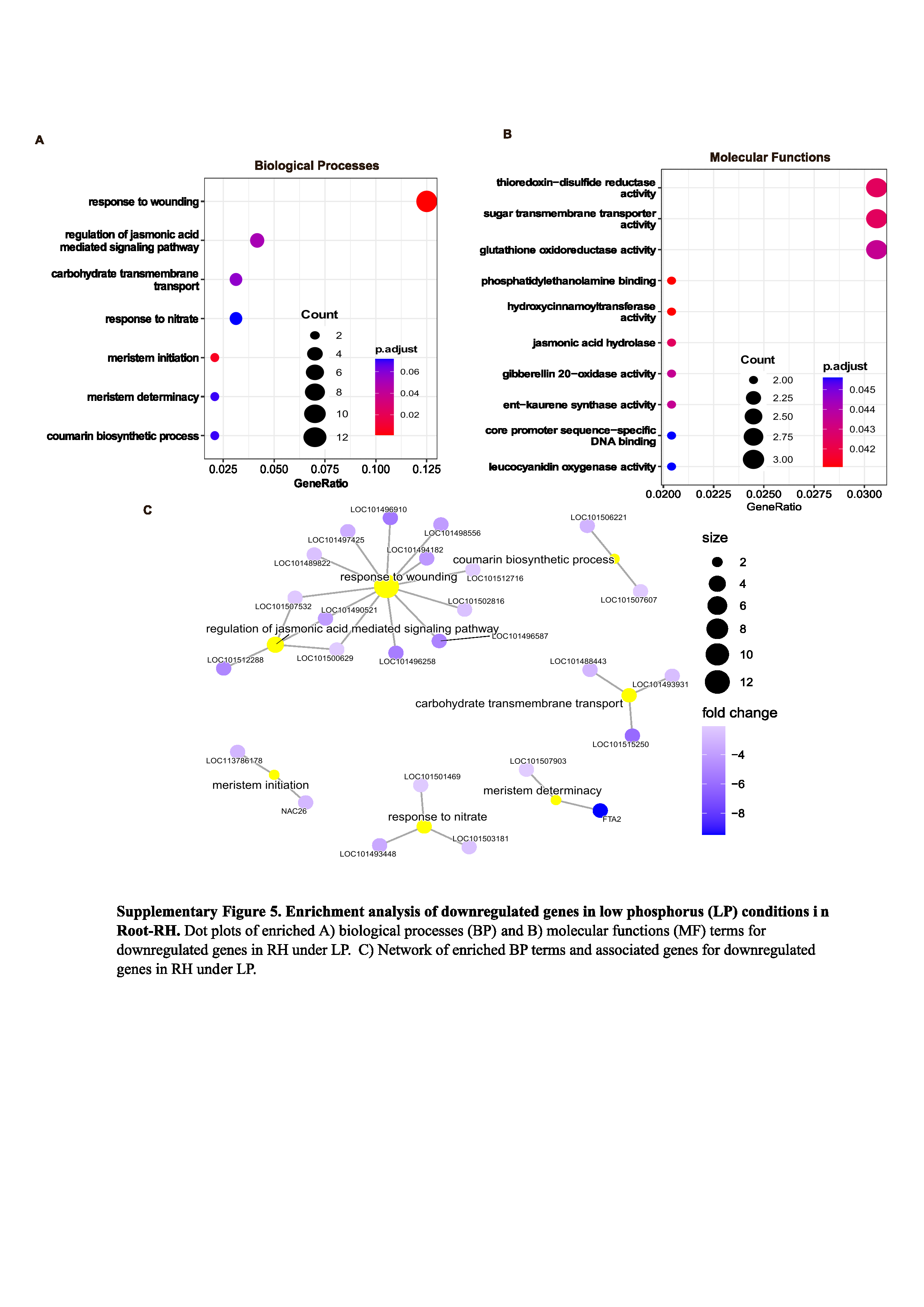

Supplement: Supplementary file 6 [file Image_5.TIFF]

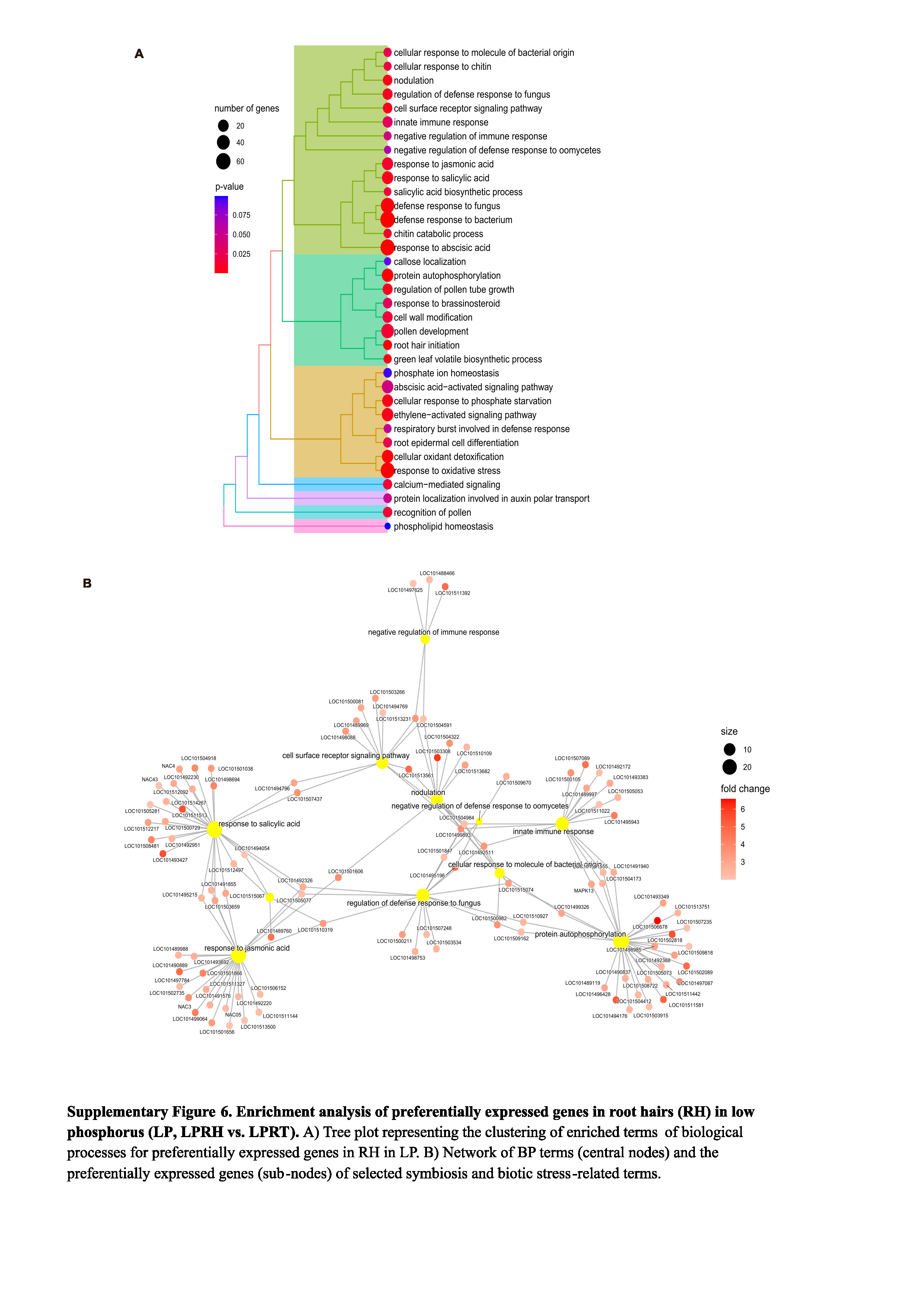

Supplement: Supplementary file 7 [file Image_6.TIFF]

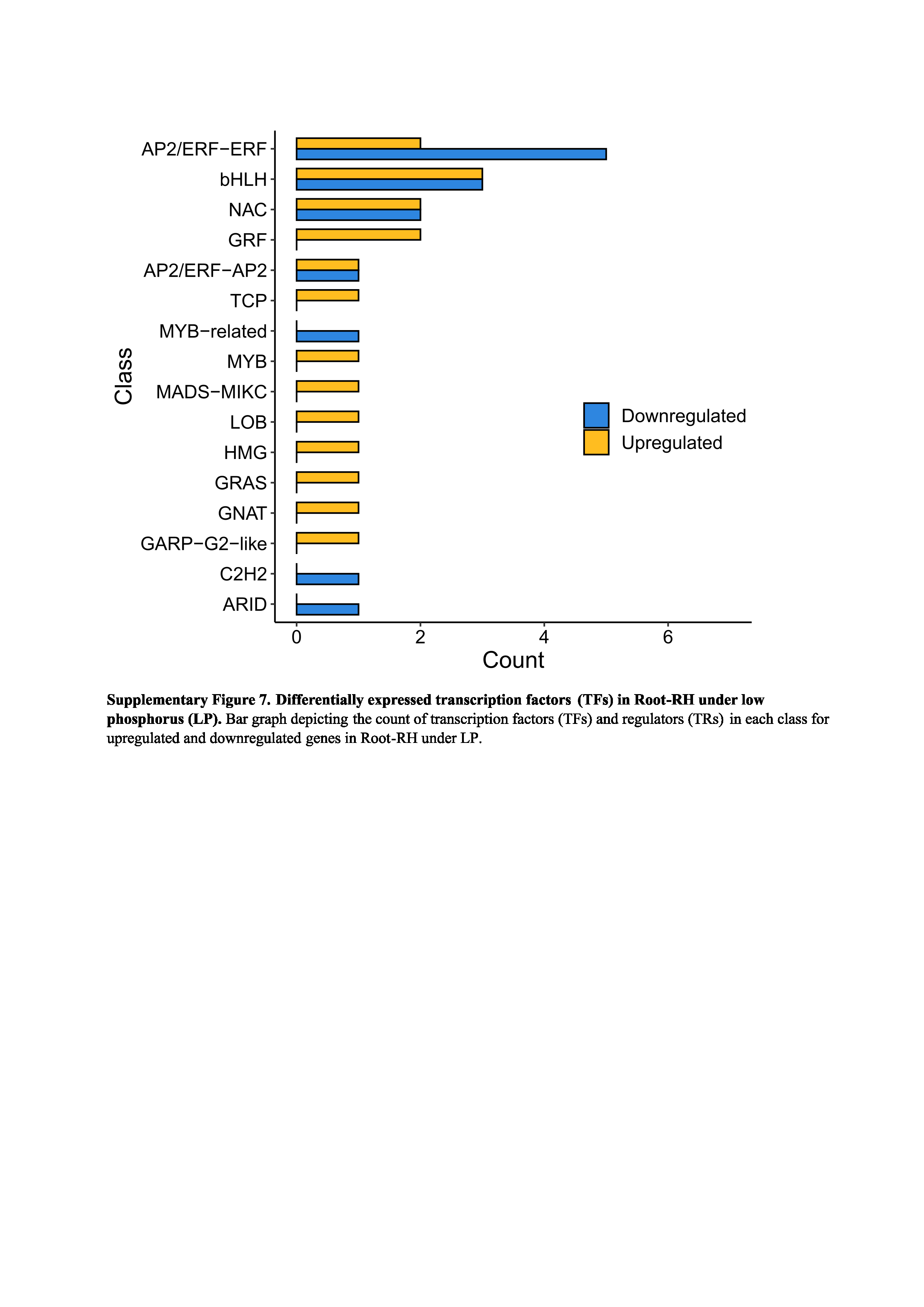

Supplement: Supplementary file 8 [file Image_7.TIFF]

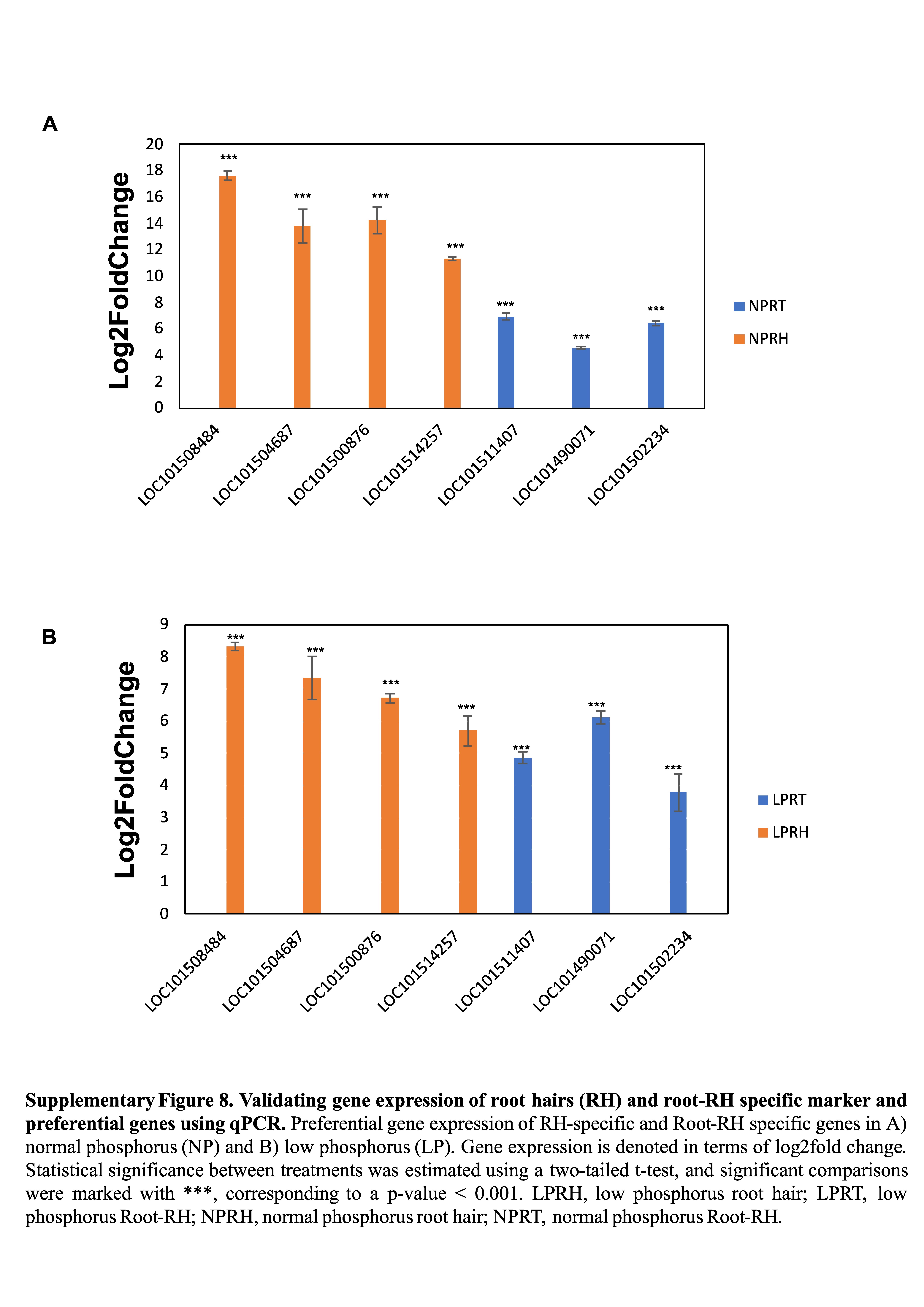

Supplement: Supplementary file 9 [file Image_8.JPEG]
